# POEM, A 3-dimensional exon taxonomy and patterns in untranslated exons

**DOI:** 10.1186/1471-2164-9-428

**Published:** 2008-09-20

**Authors:** Keith Knapp, Ashley Chonka, Yi-Ping Phoebe Chen

**Affiliations:** 1 Faculty of Science and Technology, Deakin University, Victoria, Australia; 2 Australia Research Council (ARC) Centre of Excellence in Bioinformatics, Australia

## Abstract

**Background:**

The existence of exons and introns has been known for thirty years. Despite this knowledge, there is a lack of formal research into the categorization of exons. Exon taxonomies used by researchers tend to be selected ad hoc or based on an information poor de-facto standard. Exons have been shown to have specific properties and functions based on among other things their location and order. These factors should play a role in the naming to increase specificity about which exon type(s) are in question.

**Results:**

POEM (Protein Oriented Exon Monikers) is a new taxonomy focused on protein proximal exons. It integrates three dimensions of information (Global Position, Regional Position and Region), thus its exon categories are based on known statistical exon features. POEM is applied to two congruent untranslated exon datasets resulting in the following statistical properties. Using the POEM taxonomy previous wide ranging estimates of initial 5' untranslated region exons are resolved. According to our datasets, 29–36% of genes have wholly untranslated first exons. Untranslated exon containing sequences are shown to have consistently up to 6 times more 5' untranslated exons than 3' untranslated exons. Finally, three exon patterns are determined which account for 70% of untranslated exon genes.

**Conclusion:**

We describe a thorough three-dimensional exon taxonomy called POEM, which is biologically and statistically relevant. No previous taxonomy provides such fine grained information and yet still includes all valid information dimensions. The use of POEM will improve the accuracy of genefinder comparisons and analysis by means of a common taxonomy. It will also facilitate unambiguous communication due to its fine granularity

## Background

The task of building an exon taxonomy is complex, because exons exist in multiple information dimensions. Unfortunately most exon taxonomies are either in one dimension (which is information poor) or they are incomplete. The protein oriented exon monikers (POEM) is based on dimensions known to reflect statistical features and attempts to include every possible exon category.

We developed the POEM, which can be used by all genefinders and other taxonomical functions to serve two purposes. The first is to ease prediction comparison between genefinders by means of unambiguous communication. If the predictions of all genefinders use the same taxonomy there can be no doubt as to what is predicted. Furthermore, one might as well employ an information rich instead of an information poor taxonomy. The second purpose is to investigate patterns of exons. Although exons are routinely measured, spliced and mutated little research has been done to elucidate the roles of different exon types. Given its fine granularity POEM gives the researcher the ability to analyze distinct inter-exon relationships and to aggregate POEM categories to determine more traditional statistics of a dataset.

Most exon taxonomies use one of two methods (and rarely both). The first method is to classify an exon based on its ordinal position in relation to other exons. The later method categorizes exons based on their position relative to the coding sequence (CDS) boundaries. The general problem is that either the method used is uninformative or is not implemented completely.

We call the most common ordinal taxonomy FILS (which stands for First, Internal, Last or intronlesS). In its most typical form FILS denotes the global position of an exon (namely those mentioned above), but gives no indication as to which region (5'UTR, CDS, 3'UTR or a combination thereof) the exon occurs in. FILS does not state where within a region an exon is located, and it vaguely hints at the number of exons around it. FILS is used in most genefinders [1-3]. A generalization of FILS, a two category taxonomy has been used by Brunak [4]. This method identified exons either as internal or terminal thus providing even less information.

Some automated genefinders such as those using hidden Markov models implement state transition diagrams which are in-and-of-themselves rather detailed taxonomies. For a protein oriented exon taxonomy to be complete it must describe partially and wholly untranslated exons (henceforth uexons). Despite the inclusion of untranslated regions in many hidden Markov model genefinders the resultant output data does not clearly segregate coding from non-coding exons. Furthermore many of the genefinders represent a complex untranslated region with a single state [1,3,5]. The latest versions of AUGUSTUS however do implement multiple untranslated region states while providing more comprehensive output [6].

Genlang is a genefinder where exons and other sub-genetics sequences are eventually categorized into one of ten categories [7]. Their method is based upon a linguistic understanding of the gene structure, and the category names are qualitative and give a brief description of the function that sub-genetic sequence plays. It gives little information regarding an exon's position in relation to others. The "predicted exon taxonomy" of Knapp and Chen identified 13 classes of predicted exons [8]. The intention of their taxonomy however is to evaluate genefinder accuracy versus a known true dataset and to measure the effect of modifications to genefinding software [8].

The z12 [9] taxonomy was the first to combine the FILS dimension with either the region or the CDS boundary dimension. The z12 merges two dimensions and laid the groundwork for merging multiple dimensions with unambiguous category names. Unfortunately z12 is not complete and some experimentally verified exons cannot be categorized according to it. These shortcomings spurred the need for a new and more thorough exon taxonomy. To compensate, we originally added four new classes: 5texon, 3texon, 5tuexon, and 3utexon (so named according to the original nomenclature). Furthermore the iu-exon and intronless taxons were decomposed into region or CDS boundary specific classes [10]. Additional analysis revealed that all dimensions were still not adequately characterized and thus the POEM (described below) was constructed.

The POEM taxonomy incorporates up to three dimensions of information: global position, regional position and region. It unambiguously categorizes every exon type associated with a protein coding gene, both those which have been demonstrated experimentally and those which are only biologically and theoretically legitimate. POEM combines previous methods thereby leading to a more rich nomenclature. Any of the more coarse grained taxonomies can be calculated from POEM style results making it "backwards compatible" with its predecessors. The fine granularity of categorization has two derived functions. First, data in the POEM format are in a state which facilitates inter-exon analysis. Secondly, ambiguity is reduced thus easing communication regarding exactly which exon category is in question. In the next section we provide full details of the POEM and two datasets in which every sequence contains a uexon. The subsequent section applies the POEM to the datasets and elucidates statistical relationships.

## Methods

This section presents the details of the POEM and the two datasets (TUTR and EID) to which POEM is applied. The TUTR dataset was extracted and constructed by the authors while the EID is a large subset of an existing database [11,12]. This section concludes with a comparison and contrast of the datasets. We assert that TUTR is essentially congruent to EID, thus TUTR is representative of the uexon distribution in human DNA databases.

### POEM (Protein Oriented Exon Monikers)

**Figure 1:**
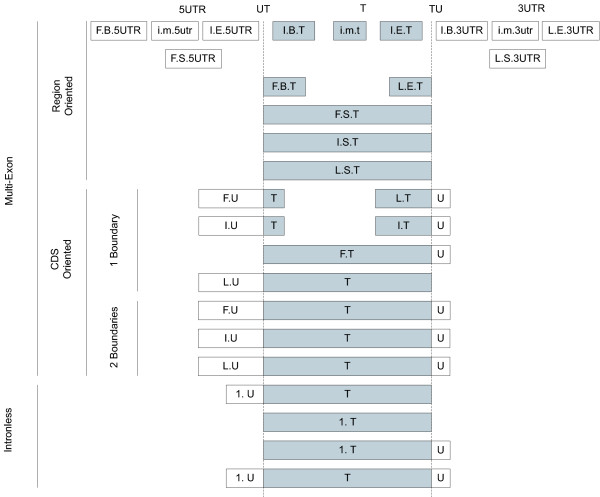
**The 29 exon categories in the POEM taxonomy**. The vertical lines to the left indicate to which component(s) an exon belongs. The regions and CDS boundaries appear across the top of the diagram. The dashed vertical lines underneath "UT" and "TU" indicate the CDS boundaries. Each box or combination of adjacent white and shaded boxes represents one of the 29 exon categories. The translated region is darkened to aid visual demarcation from untranslated regions. An exon's moniker (or category name) is the combination of letters found within an exon. Dimension values are separated by periods (despite any CDS boundary). Lower case category names represent exons which can occur multiple times in the same protein coding gene; whereas all upper case monikers indicate exon categories that occur 0 or 1 times in a given protein coding gene. Space between two exon categories is to be understood as intronic. Place-holding exons are not displayed. Place-holding exons are those required by the taxonomical constraints to precede or follow a particular exon category. For example, internal exons (those whose monikers commence with an "I" or "i") can only exist if both preceded and followed by another exon.

Given the lack of thorough protein oriented exon taxonomies the need for a new categorization method was obvious. The 29 categories of the POEM are displayed in Figure 1; Table 1 lists each exon category by name and gives a brief description. Despite the relatively long length of Table 1 the monikers are learned and used relatively quickly. The POEM taxonomy is divided into multi-exon genes and intronless-genes (also known as single exon genes [13]). Multi-exon genes are further sub-divided into region- and CDS- oriented exons. POEM therefore consists of three main components: intronless genes, CDS-oriented exons and region-oriented exons.

**Table 1:** POEM Exon Category Summary

Moniker	Brief	Description
F.B.5UTR	First, Beginning, wholly in 5' UTR	The most 5' exon in a gene, which is also the beginning of multiple exons wholly within the 5' UTR.
F.B.T	First, Beginning, wholly in the translated region	The most 5' exon in a gene, which is also the beginning of multiple exons wholly within the translated region.
F.S.5UTR	First, Intronless, wholly in 5' UTR	The most 5' exon in a gene, which is also the only exon in the 5' UTR.
F.S.T	First, Intronless, wholly in the translated region	The most 5' exon in a gene, which is also the only exon in the translated region.
F.TU	First, Translated-Untranslated	The most 5' exon in a gene, which also spans the entire translated region and the 3' CDS boundary.
F.UT	First, Untranslated-Translated	The most 5' exon in a gene, which begins in the 5' UTR and ends in the translated region.
F.UTU	First, Untranslated-Translated-Untranslated	The most 5' exon in a gene, which begins in the 5' UTR and ends in the 3' UTR.
I.B.3UTR	Internal, Beginning, wholly in 3' UTR	A non-terminal exon that is also which is also the beginning of multiple exons wholly within the 3' UTR.
I.B.T	Internal, Beginning, wholly in the translated region	A non-terminal exon that is also which is also the beginning of multiple exons wholly within the translated region.
I.E.5UTR	Internal, End, wholly in 5' UTR	A non-terminal exon that is also which is also the end of multiple exons wholly within the 5' UTR.
I.E.T	Internal, End, wholly in the translated region	A non-terminal exon that is also which is also the end of multiple exons wholly within the translated region.
i.m.3utr	Internal, Middle, wholly in 3' UTR	A non-terminal exon that is also which is surrounded by exons which are wholly in the 3' UTR.
i.m.5utr	Internal, Middle, wholly in 5' UTR	A non-terminal exon that is also which is surrounded by exons which are wholly in the 5' UTR.
i.m.t	Internal, Middle, wholly in the translated region	A non-terminal exon that is also which is surrounded by exons which are wholly in the translated region.
I.S.T	Internal, Intronless, wholly in the translated region	A non-terminal exon that is also which is also the only exon in the translated region.
I.TU	Internal, Translated-Untranslated	A non-terminal exon that is also which begins in the translated region and ends in the 3' UTR.
I.UT	Internal, Untranslated-Translated	A non-terminal exon that is also which begins in the 5' UTR and ends in the translated region.
I.UTU	Internal, Untranslated-Translated-Untranslated	A non-terminal exon that is also which begins in the 5' UTR and ends in the 3' UTR.
L.E.3UTR	Last, End, wholly in 3' UTR	The most 3' exon in a gene, which is also the end of multiple exons wholly within the 3' UTR.
L.E.T	Last, End, wholly in the translated region	The most 3' exon in a gene, which is also the end of multiple exons wholly within the translated region.
L.S.3UTR	Last, Intronless, wholly in 3' UTR	The most 3' exon in a gene, which is also the only exon in the 3' UTR.
L.S.T	Last, Intronless, wholly in the translated region	The most 3' exon in a gene, which is also the only exon in the translated region.
L.TU	Last, Translated-Untranslated	The most 3' exon in a gene, which begins in the translated region and ends in the 3' UTR.
L.UT	Last, Untranslated-Translated	The most 3' exon in a gene, which begins in the 5' UTR and ends in the translated region.
L.UTU	Last, Untranslated-Translated-Untranslated	The most 3' exon in a gene, which begins in the 5' UTR and ends in the 3' UTR.
1.T	Intronless, Translated	An intronless gene exists only in the translated region.
1.TU	Intronless, Translated-Untranslated	An intronless gene which also spans the entire translated region and the 3' CDS boundary.
1.UT	Intronless, Untranslated-Translated	An intronless gene which also spans the 5' CDS boundary and the entire translated region.
1.UTU	Intronless, Untranslated-Translated-Untranslated	An intronless gene which begins in the 5' UTR and ends in the 3' UTR.

### Components

**Figure 2:**
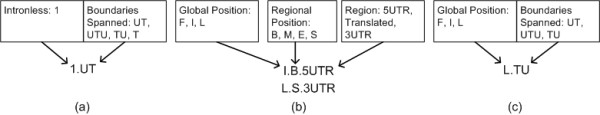
**The dimensions of exon categories.** Frame (a) identifies the four types of intronless exons. The digit before the period indicates an intronless gene with only one "exon". The dimension value following the period represents which CDS boundaries are spanned. Frame (b) states the 3 parts of a regional exon. The leftmost value states the exon's position with respect to all other exons in the same gene; the middle value indicates the exon's position within its region (which is represented by the rightmost value). Frame (c) represents a CDS-oriented exon, by first stating its global position followed by an indicator of which CDS boundaries are spanned.

Each of these components is introduced along with the nomenclature used. The intronless component consists of four categories: 1.UT, 1.T, 1.TU and 1.UTU; (Figure 1 bottom) each of their names is comprised of two parts (Figure 2a). The digit in their name indicates there is only one "exon" in the protein coding gene currently being considered, thus the entire translated region is always included. The suffix of an intronless category name (that which follows the period) indicates which CDS boundary (if any) is spanned. The four options for CDS boundary spanning are: (1) the 5' CDS, (2) no boundary, (3) the 3' CDS or (4) both. For example 1.UT exons enter the 5'UTR; while 1.UTU spans the translated region and enter both UTRs.

There are 16 region-oriented exons and the name of each consists of three parts: a prefix, a midfix and a suffix each separated by a period (Figure 2b). The prefix indicates the global ordinal position of the current exon considering all exons within the entire transcript. Valid values for the prefix are F (first), I (internal) and L (last). The midfix states the ordinal position of the exon, but only within its region, not globally. The four values for a midfix are: B (beginning), M (middle), E (End) or S (intronlesS). The suffix indicates in which region the exon resides. There are three regions in the POEM: the 5' untranslated region (5UTR), the translated region and the 3' untranslated region (3UTR). An F.B.T exon is thus the very first exon in a gene, as well as the beginning exon among those in the translated region; whereas and I.B.T is the first inside the translated region but internal in relation to all other exons in the gene.

Nine CDS-oriented categories exist, each CDS-oriented exon spans either one or two CDS boundaries. The name of a category consists of two parts: the global position (as with region-oriented exons), followed by the spanned CDS boundaries (as indicated in Figure 2c). CDS-oriented exons have no regional position as per definition they are in at least two regions. A F.UTU for example is the first exon of a multi-exon gene and it spans both CDS boundaries. It will be followed by at least one untranslated exon in the 3' UTR. An L.TU exon would be the very last in a gene and it may span part-of or the entire translated region. It does not exist in the 5' UTR, but terminates in the 3UTR.

There are some constraints imposed on the monikers by this taxonomy. All exon categories are constrained by the existence of a global position dimension, except for intronless genes. All genes with multiple exons must have a first and last exon. There are seven first and seven last exon categories. There is no further constraint imposed by the taxonomy indicating which first and last exon is paired with as long as they are biologically valid. As an example, the existence of a UTU obviously excludes the existence of a UT or TU in the same gene, and vice-versa. Similarly some CDS-oriented exons exclude certain region-oriented exons from existence in the same gene. An I.UTU would exclude any region-oriented exon in the translated region, however does not express any information regarding the presence of exons in either UTR.

Other constraints exist in the regional position dimension; each beginning exon must have an end exon. The beginning and end exons in either untranslated region can only co-exist with one other category. F.B.5UTR always occurs in conjunction with I.E.5UTR; likewise an I.B.3UTR always has an L.E.3UTRs. Lastly, there are two beginning and two end exons in the translated region, either beginning translated exon can occur with either end translated exon.

### Properties

The POEM places each exon in up to three dimensions: global position, regional position and region (region-oriented or CDS spanning) as applicable. The global position uses three of the four FILS positions (excluding intronless). The regional position uses all of the FILS values, but its meaning is restricted to a particular region. Although regional position uses the FILS concept, it implements different letters (B, M, E and S) to prevent confusion of the global and regional position values.

A putative fourth dimension for POEM is count; capitalized category names indicate an exon count of either 0 or 1 for any given gene; whereas lowercase monikers may exceed a count of one. Only 3 categories may have a count greater than one, i.m.5utr, i.m.t, and i.m.3utr, and thus only these are ever in lower-case.

Two additional factors support the existence of the POEM taxonomy. The first is that unlike its predecessors it seems to cover all valid protein related exon categories. As we have previously identified multiple experimentally validated exon types that would not fit in any other multiple dimension exon taxonomy [10] this is sufficient justification. POEM however is not designed to categorize exons in non-translated genes. The second reason is that the selected dimensions of POEM are supported by prior research.

It is important to differentiate between intronless and multi-exon genes as the latter can be alternatively spliced and failure to be aware of this may limit proper analysis. Furthermore, intronless genes display higher stability (that is a greater change in minimum free energy) than multi-exon genes [10] so thermodynamic research into exon structure and composition may need to be adjusted appropriately. Intronless gene categories tend to be associated with specific lengths and thus categorization immediately indicates sequence length (Knapp and Chen, submitted). The choice to include both CDS-oriented exons and region-oriented exons is based upon the reasoning that until recently no genefinders included either of these categories in their exon definition models. The UTR region of CDS-oriented exons were simply truncated at the CDS boundary and the untranslated portions were not even identified using genefinders. The categorization of CDS-oriented exons and region-oriented exons is necessary to identify all exons and relationships within a protein coding gene. When comparing and contrasting region-oriented exons a number of statistical properties emerge; first, 5'UTR exons have a higher CG content than translated exons [14,15]. Second, translated exons are easier to identify than those in the UTR, probably due to having a higher percentage of canonical splice site boundaries. Eden and Brunak have also show that the 3' ends of UTRs have a distinct compositional and positional bias [15].

In comparing the two untranslated regions, we show that uexon genes are 5' heavy compared to 3' exons (below), thus confirming that the 5'UTR needs to be information rich for transcription initiation. In contrast, others have shown that the 3'UTR is heavy in translational regulatory features [16]. Finally, expression in plants has been shown to be length dependent on the 5' UTR exons [16].

The global position (implemented with FILS) is the de facto standard for exon taxonomies and is essentially the bare minimum taxonomy with any meaningful information. Most researchers expect this information. Furthermore, it has been shown that the first exon is important to transcription regulation, while the last exon tends to have a low CpG content [17]. The use of regional position instantly provides multiple levels of information. For example, F.B.5UTR exons are known to have high GC content [17]. In addition to indicating location, the regional position value also indicates a minimum of how many other exons exist in the region. A middle exon for example automatically denotes the presence of at least 2 more exons, one upstream and one downstream.

### POEM Summary

It should be stated that the POEM is not a gene taxonomy; it does not address the promoter or poly-adenosine regions or the logical regions therein. Furthermore it does not categorize RNA only encoding genes. Introns and non-coding non-exonic regions likewise are not within its scope.

The POEM is thorough in that every dimension is applied to all other dimensions; due to logical contradictions however numerous redundancies are eliminated. As known and frequently used dimension are included there is no loss of information as compared to not having multiple dimensions. POEM formatted results can be aggregated and thus more traditional coarsely grained taxonomic statistics are also computationally accessible. Finally, the POEM includes biologically real exons. Unlike the states used in some automated genefinders [3,5], the POEM attempts to identify every possible protein oriented exon type, especially those which span CDS boundaries. Some categories such as L.UT have yet to have experimentally verified exons, but with the existence of L.UTU exons the option for their existence is undeniable.

### Datasets

Originally one dataset was built for exon relationship analysis, after a thorough filtering process the TUTR dataset was finished. Upon review we found it practical to compare TUTR with a larger and established dataset to verify exon relationships and conclusions drawn. We selected the exon-intron database [11,12] over others [18] because it is larger and more current. The EID has 26,319 human FASTA entries including intronless genes from GenBank release 149; whereas ExInt has 10,423 human genes from GenBank release 122. This section describes the process of building TUTR and our filtering applied to EID.

### TUTR (The UnTRanslated)

We started by extracting all human sequences annotated as "complete cds" from GenBank's release 151 of the Entrez Nucleotide website [19] with an entry date prior to Jan. 1st 2006 (61,373 sequences in total). We then filtered out all sequences matching the following criteria: (1) sequences derived from mRNAs (totalling 49,036 sequences), (2) alternatively spliced genes, (3) GenBank records that contain the "SEGMENTS" keyword, (4) entries that did not contain the keyword "CDS", (5) genes not containing uexons or (6) records with ambiguous features (using "<" or ">") in a CDS or exon entry [13]. In order to eliminate sequences with high similarity, this resulting dataset was then analyzed with clustalW [20] version 1.8, 38 redundant sequences were removed that had a score of 90 or higher. The resulting dataset was 348 sequences each with only one known transcript and containing at least one uexon. TUTR348 has 2,619 exons of which 847 are uexons. The TUTR dataset [see Additional file 1] is available at BMC Genomics.

### EID (Exon-Intron Database)

**Figure 3:**
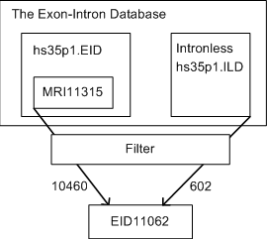
**The process of building the EID dataset.** The Exon-Intron Database contains dozens of files with the human exons and introns stored in hs35p1.EID and hs35p1.ILD.

The Exon-Intron Database [11,12] contains all the human exons and introns from GenBank release 149 in the file hs35p1.EID. The file MRI11,315  is the published non-redundant subset of the Exon-Intron Database. We built our comparison database, EID, by combining the result of MRII11,315 with the separate intronless dataset hs35p.ILD, as shown in Figure 3.

MRI11,315 actually only contained 11,314 sequences. We double checked the MRI sequences against the original Exon-Intron Database using the value of the "gene=" field in the header and found 105 entries in MRI that were not seemingly from the EID. We removed these 100 sequences along with those in MRI that did not contain uexons (726) or those which contained contradictory exon or CDS boundaries (23), resulting in 10,460 non-redundant records.

The Exon-Intron Database's intronless sequences are held separate from its multi-exon genes. We filtered out all intronless sequences not having uexons and redundant genes with a clustalW score > 90. We combined these resultant 602 intronless sequences with the multi-exon genes to obtain 11,062 sequences (henceforth EID) consisting of 101,718 exons of which 27,043 are uexons.

## Results and discussion

Upon completion of the two datasets software algorithms were developed to categorize every exon into one of the 29 POEM categories. The following two subsections will discuss congruency between TUTR and EID followed by the elucidation of exon patterns.

### Congruent Dataset

The distribution of sequences, components and exon categories will be presented in this section supporting our assertion that EID and TUTR are congruent in many areas. As EID is essentially the uexon containing genes taken directly from GenBank, the fact that TUTR348 is congruent, yet smaller is highly beneficial when a researcher is facing high computational loads. Three examples of such situations are: (1) when computations have an exponential or factorial (BLAST all against all) increase in time as a factor of the sequence count, (2) when computation time increases exponentially as a factor of sequence length e.g. RNA or protein folding [21-23] or (3) those in which an extensive literature search is expected to follow computations. In comparing and contrasting the two datasets a difference factor of approximately-thirty occurs numerous times. For example, the average multiplication factor (the mean average of the bottom rows in tables 2 and 3) between each exon category for both datasets is 36 (with a standard deviation of 36). The EID dataset contains 31 times more genes than TUTR (11062 compared to 348). As shown in the bottom row of Table 2, the 5'UTR categories display a tight cohesiveness with factors between 25 and 29; obviously just slightly below 30. The approximately-thirty relationship is also seen in the CDS-oriented exons. Despite one slightly high value (I.TU) the exon categories are quite close to the value thirty and average out to 36. Many of the wholly translated exons (Table 2) have factor values close to thirty; the mean average being 36. The exon category L.S.3UTR (Table 2) has a multiplication factor value at 37.95 just barely over the average for all categories. Such consistencies are highly indicative of the two datasets being not necessarily exact, but congruent.

**Table 2:** The distribution of region oriented exons

	5UTR				T								3UTR			
			
	F.B.5UTR	i.m.5utr	I.E.5UTR	F.S.5UTR	F.S.T	F.B.T	I.B.T	i.m.t	I.E.T	L.E.T	I.S.T	L.S.T	I.B.3UTR	i.m.3utr	L.E.3UTR	L.S.3UTR
			
TUTR Category Count	33	24	33	97	0	11	230	1243	228	13	46	1	3	4	3	20
% of all TUTR exons	0.013	0.009	0.013	0.037	0	0.004	0.088	0.475	0.087	0.005	0.017	0	0.001	0.002	0.001	0.008
EID Category Count	886	696	886	2446	166	912	7506	56731	8275	143	912	30	347	516	347	759
% of all EID exons	0.009	0.007	0.009	0.024	0.002	0.009	0.074	0.561	0.082	0.001	0.009	0	0.003	0.005	0.003	0.008
Factor	26.848	29	26.848	25.216	n/a	82.909	32.777	45.64	36.454	11	20.267	30	115.667	129	115.667	37.95

The top row identifies which region (5' UTR, the translated region or the 3' UTR); while ths second row states the POEM exon category. For each category there are five rows of data displayed: (1) The exon count in the TUTR dataset, (2) The percentage of all exons in TUTR, (3) The count in the EID dataset, (4) The percentage of all exons in EID and (5) The multiplication factor separating the two TUTR and E counts.

**Table 3:** The distribution of CDS oriented exons

	F.UT	F.UTU	F.TU	I.UT	I.UTU	I.TU	L.UT	L.UTU	L.TU
	
TUTR Category Count	196	3	0	109	0	20	0	19	284
% of all TUTR exons	0.075	0.001	0	0.042	0	0.008	0	0.008	0.108
EID Category Count	6050	0	0	3249	0	1079	0	0	9180
% of all EID exons	0.06	0	0	0.032	0	0.011	0	0	0.091
Factor	31	-	-	30	-	54	-	-	33

The top row identifies the CDS oriented POEM categories. For each category there are five rows of data displayed: (1) The exon count in the TUTR dataset, (2) The percentage of all exons in TUTR, (3) The count in the EID dataset, (4) The percentage of all exons in EID and (5) The multiplication factor separating the two TUTR and EID counts.

**Figure 4:**
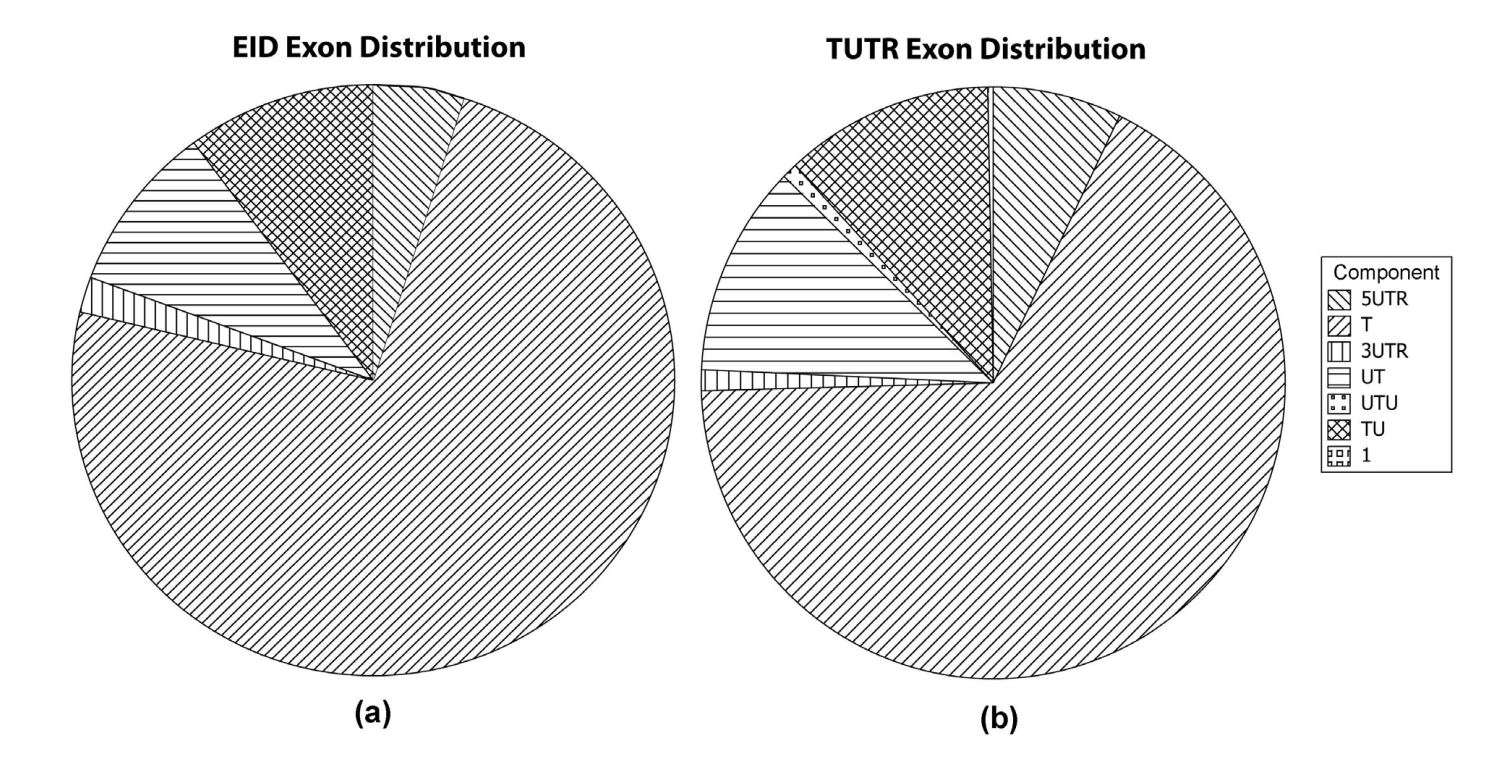
**The distribution of exon components as a fraction of all exons.** Figure (a) contains the distribution for EID exons. Figure (b) the distribution for TUTR exons. The components shown are all disjoint and include all exons in the dataset. The components are based on region, CDS boundary or intronless genes. In the legend T stands for translated exons, 1 indicates intronless genes and the remaining symbols for the named region or CDS boundary spanning type.

Another form of congruency can be seen at the component level. In figure 4, the exon distributions for the major components are highly similar between TUTR and EID. The largest percentage of all exons is the translated exons accounting for approximately 75%. The next two largest categories are the UT and TU exons covering ~10% and 9% of the datasets, respectively.

Of course not all exon categories hold to the approximately-thirty rule. The 3UTR exons that are part of multi exon genes have factors greater than 100 and the intronless genes (Table 4) are either extremely low or high. The TUTR dataset was not built with the intention of being congruent to another so it is not expected that all categories will be so.

**Table 4:** The distribution of intronless genes

	1.UT	1.T	1.TU	1.UTU
	
TUTR Category Count	3	0	1	4
% of all TUTR exons	0.375	0	0.13	0.5
EID Category Count	18	0	2	582
% of all EID exons	0.03	0	0	0.967
Factor	6	-	2	146

The top row identifies the Intronless POEM categories. For each category there are five rows of data displayed: (1) The exon count in the TUTR dataset, (2) The percentage of all exons in TUTR, (3) The count in the EID dataset, (4) The percentage of all exons in EID and (5) The multiplication factor separating the two TUTR and EID counts.

Four categories have an exon count of zero in both datasets (1.T, F.TU, I.UTU and L.UT in Table 2). By definition 1.T genes have no uexons and therefore will not occur in the datasets as they focus on protein coding genes that contain uexons. The remaining three categories have the following two properties in common: (1) they are all CDS-oriented exons and (2) they span the entire translated region. Despite no instances of these exons being found it does not preclude their existence. In fact given that F.UTU and L.UTU exons have been shown to exist (Table 3) we believe it is likely that F.TUs or L.UTs may be found, perhaps in a less strictly filtered dataset. We were initially surprised that no I.UTU exons were identified. Given that numerous exons span the entire translated region I.UTUs were expected to have one of the higher UTU counts. A number of options may account for their non-presence: (1) the genes containing I.UTUs were coincidentally removed during filtering (of highly homologous sequences), (2) they just don't exist, (3) insufficient useful information has evolved around the ends of other .UTUs to convert them to I.UTUs or (4) the practical role played by a terminal exon was not of sufficient importance to help it overcome selection thus I.UTU became a degenerate in another category.

EID has 166 F.S.T exons (Table 1) where as TUTR has none. Using the 30:1 ratio TUTR would be expected to have at least 5 F.S.T exons. By definition F.S.T must have at least one exon partially in the 3UTR, thus genes with F.S.T would not have been filtered out for not having an untranslated region. The reason for their non-existence is currently elusive. Similarly TUTR has exons in categories EID does not (F.UTU and L.UTU in Table 3), according to the 30:1 ratio EID, should have about 90 F.UTUs and 630 L.UTUs. None of these missing exons is unimaginable given that 1.UTU in EID has over 582 entries (Table 3). If future exon categorization projects fail to identify these exon types, it may be indicative of some functional constraint preventing their existence based upon their location and/or other properties.

No two independently derived datasets will ever be exactly the same in each category, but TUTR and EID display remarkably close similarities in sequence count, region-oriented exon and CDS-oriented exon distribution and the majority of POEM categories. This congruency aids validation of TUTR348 as a representative distribution of the uexon containing sequences in the human genome. In particular, scientists studying uexons will find TUTR's size beneficial when facing compute intensive tasks.

### Exon Patterns

Previous estimates of the number of human genes with first wholly untranslated exons have varied between 2% and 40% [24,25]. The EID contains 3332 genes where the first exon is wholly untranslated (886 F.B.5UTR and 2446 F.S.5UTR), meaning that 29% of the original MIR11,314 dataset contains a first wholly untranslated exon. This is vastly larger and somewhat smaller than the two estimates given above. According to TUTR 36% of human genes contain an initial wholly non-coding exon. Sufficient information is not given by Davuluri [24] as to how they apply their 40% value to the entire human genome from a sample of 2100 genes, especially since their work was published on or before the release of the draft sequences of the human genome. Computational identification of first exons is very important, in particular those which are followed by multiple exons which are also wholly within the untranslated region. It can be difficult to build primers which lead to sufficient duplication of the terminal regions of genes, thus effective annotation of these exons by computational means can save researchers invaluable resources.

In comparing the 5UTR to 3UTR exon distribution there is a clear indication that uexon genes are 5' heavy, that is there are many more 5UTR exons than 3UTR. As displayed in Table 2 and Figure 4, the EID 5' UTR exons outnumber their 3' counterparts by more than double. The TUTR 5' exons outweigh the 3' exons by more than a factor of four. A likely reason for the 5' heaviness would be to ensure sufficient specificity for binding of appropriate expression regulatory factors. Furthermore this indicates that relatively less information is necessary for transcription or translation termination. Another reason could simply be that the 3'UTR is not studied as much [26] and thus fewer exons and their associated binding sites have been identified.

In Table 3, as the global position for .UT exons progresses from F to I to L the count decreases. Likewise the count decreases for the .TU exons when the global position progresses in the opposite order. Is there a reason why there should be more F.UTs than I.UTs? Or more L.TUs than I.TUs? The F.UTs outnumber I.TUs by at least 2:1 and the L.TUs against I.TU 9:1. The I.UTs and I.TUs require more complex information (UTR exons) to be added to their terminals to regulate expression and this has not occurred, thus these proportions are not surprising.

Over half of all sequences have a F.UT as their first exon; specifically TUTR 55% and EID 56%. On the 3' end ~80% of all genes in MRI11,314 terminate with an L.TU exon. Obviously there is a high propensity for uexon sequences to terminate with CDS-oriented exons and not just wholly untranslated exons. Furthermore CDS-oriented exons do not display the same 5' heaviness as the region-oriented exons. When comparing UTs vs. TUs their total counts are about the same with ~300 in TUTR and ~10,000 in EID (note again the 30:1. ratio).

Despite all the possible exon combinations, CDS-oriented exons are highly influential. A visual analysis of the exon distribution showed that three disjoint exon patterns (Table 5) exist comprising 70% of all multi-exon genes. The most frequently of which is, F.UT, I.B.T, L.TU comprises 38% of all uexon sequences. It will be interesting to investigate these and other exon patterns further to identify functional relationships such as: if exons may function as building blocks which are re-used in other genes, to improve automated genefinder prediction by incorporating exon relationships.

**Table 5:** Three patterns of untranslated exons

Exon patterns			TUTR	EID
F.UT	I.B.T	L.TU	134	4446
F.S.5UTR	I.UT		82	2383
F.B.5UTR	I.UT	L.TU	24	724
			
Percentage of multi-exon genes:			0.694	0.7221

The right hand column specifies the count for each exon pattern (left).

## Conclusion

In this work we describe a taxonomy for protein related exons, POEM, which is biologically and statistically relevant. No previous taxonomy has provided such fine grained information and yet included all possible dimension combinations. Use of POEM will improve comparative analysis of genefinder accuracy through the use of a consistent taxonomy. It will also facilitate unambiguous communication due to its fine granularity. We applied this taxonomy to two congruent uexon datasets differing in size by approximately a factor of thirty. The smaller of the two will be advantageous to those with heavy computational expectations.

Using this taxonomy we identified statistical features of uexon containing genes in two datasets. We revised the estimate of first completely untranslated exons to be between 29 – 36%. Untranslated exon genes are shown to have more 5UTR exons than 3UTR exons, while CDS boundary spanning exons are evenly distributed. CDS boundary spanning exons are also shown to be proportional to the amount of information needed to manage transcription or translation. Finally we identified three exon patterns which comprise the majority of all uexon containing genes in our datasets. Future work will include construction of a thorough non-coding taxonomy, the implementation of all POEM categories in a genefinder and the addition of multiple dimensions to POEM.

## Authors' contributions

KDK identified the ideas, implemented the concepts and wrote this paper. AC reviewed this paper. YPPC supervised the work and participated in the writing process and reviewed this paper. All authors read and approved the final manuscript.

## Supplementary Material

Additional file 1 **The TUTR348 dataset.** The data provided represent 348 human genes containing one known transcript and at least one partially or wholly untranslated exon. Click here for file
